# Facile Preparation of Cellulose Fiber Reinforced Polypropylene Using Hybrid Filler Method

**DOI:** 10.3390/polym14081630

**Published:** 2022-04-18

**Authors:** Safarul Mustapha, Jacqueline Lease, Kubra Eksiler, Siew Teng Sim, Hidayah Ariffin, Yoshito Andou

**Affiliations:** 1 Department of Biological Functions Engineering, Graduate School of Life Science and Systems Engineering, Kyushu Institute of Technology, 2–4 Hibikino, Wakamatsu, Fukuoka, Kitakyushu 808-0196, Japan; paroys_9@yahoo.com (S.M.); lease.jacqueline708@mail.kyutech.jp (J.L.); kubra.eksiler@gmail.com (K.E.); simsiewteng92@gmail.com (S.T.S.); 2 Faculty of Biotechnology and Biomolecular Sciences, Universiti Putra Malaysia (UPM), Serdang 43400, Selangor, Malaysia; hidayah@upm.edu.my; 3 Collaborative Research Centre for Green Materials on Environmental Technology, Kyushu Institute of Technology, 2–4 Hibikino, Wakamatsu, Fukuoka, Kitakyushu 808-0196, Japan

**Keywords:** cellulose nanofiber, silica, polypropylene, composite, hybrid filler

## Abstract

Dried hybrid fillers comprised of silica/CNF were successfully synthesized in ethanol/water mixed solvents at room temperature without the usage of any precursor. The as-prepared fillers were incorporated with polypropylene (PP) as a polymer matrix through a twin-screw extruder. From surface morphology analysis, the agglomeration of the silica/CNF hybrid fillers was prevented in the PP matrix and they exhibited moderate transparency, around 17.9% and 44.6% T at 660 nm. Further, the chemical structures of the polymer composites were identified by Fourier transform infrared (FT-IR) analysis. According to thermogravimetric analysis (TGA), the insertion of silica as a co-filler to the PP matrix resulted in an increase in its degradation onset temperature and also thermal stability. In addition, the mechanical properties of the PP composites also increased after the blending process with the hybrid fillers. Overall, sample PP-SS/CNF exhibited the highest tensile strength, which was 36.8 MPa, or around 73.55% compared to the pristine PP. The improvements in tensile strength were attributed to good dispersion and enhanced efficiency of the stress transfer mechanism between the silica and the cellulose within the PP matrix. However, elongation of the sample was reduced sharply due to the stiffening effect of the filler.

## 1. Introduction

A polymer matrix composite is a material consisting of a polymer matrix with a reinforcing dispersed phase. Incorporating inorganic fillers into a polymer matrix can give the composite unique properties such as rigidity, high thermal stability, good mechanical property, flexibility, and ductility [1]. Fillers with particle sizes in the 1–100 nm range are defined as nanofillers. Generally, nanofillers are categorized into three types based on their geometries: one-dimensional (1D, rod-like), two-dimensional (2D, platelet-like), and three-dimensional (3D, spherical) materials [2]. Important factors used to determine the reinforcing effect of these fillers are the polymer matrix’s properties, the nature and type of filler, concentration of polymer and filler, and particle size as well as particle distribution [1]. Moreover, the most common nanofillers include metallic nanoparticles, polyhedral oligomeric silsesquioxane, carbon nanomaterials, graphite nanoplates, silica nanoparticles, and nanocellulose [3,4,5]. These nanofillers are inserted into polymer matrixes improve the mechanical properties, gas and solvent barrier properties, thermal degradability, and chemical resistance of the polymer [6,7]. In contrast, traditional micro-fillers can lead to polymer embrittlement, loss of transparency, and loss of lightness.

Cellulose fiber has drawn considerable interest as natural filler in polymer composites due to its superior mechanical properties, eco-friendliness, processability, biodegradability, biocompatibility, low toxicity, cost savings, and improved fuel efficiency [8,9]. However, it is difficult to disperse it properly in hydrophobic polymer because of their different surface properties. Fillers with various properties are used as hybrid fillers along with reinforcing fibers to further enhance physical and mechanical properties.

Researchers have utilized more than one filler material to investigate the synergistic effect of fillers on the final properties of polymer composites [10]. Anwer et al. [11] prepared nanocomposites using epoxy resin with carbon nanofibers (CaNFs), graphene nanoplatelets (GNPs), and a hybrid combination of CaNFs/GNPs as fillers. These composites were processed with and without the use of surfactants. It was proposed that GNPs prevented agglomeration of CaNFs during the process, leading to larger particle aspect ratios in the nanocomposite. In addition, Kwak et al. [12] successfully prevented the agglomeration of CaNFs using fish-derived gelatin during the dehydration process. According to Thomas et al. [13], a hybrid filler of carbon nanotubes (CNTs)/clay enhanced dispersion through the synergism and prevented agglomeration in the composite blend. A hybrid filler can improve crosslink density, tensile strength, and tear resistance due to the large contact area between clay and CNTs.

Typically, silica/cellulose-reinforced polymer composites have been prepared using the sol-gel method. Li et al. [14] synthesized cellulose nanocrystal/silica hybrids using TEOS as the silica precursor. This hybrid material was melt-blended with ultra-high molecular weight polyethylene (UHMWPE) polymer in a twin-screw extruder. The nanocomposite showed improvement in its flexural modulus and tensile and flexural strength. Although this sol-gel method is widely used to produce hybrid fillers, the precursors, such as TEOS, are relatively expensive and contain high amounts of embedded energy [3,15].

Herein, an ethanol/water mixed-solvent method was proposed as an accessible, fast, and low-cost protocol to prepare silica particles (SiPs) with cellulose nanofibers (CNFs) without chemical modification. Ethanol is miscible with water at any ratio, and the addition of ethanol to water can easily change its physicochemical properties. The in situ nucleation and growth of silica onto cellulose occurs rapidly in an aqueous solution. However, the addition of ethanol to water can restrain the diffusion of ions and the nucleation and growth of the silica. It is well known that rapid nucleation and slow growth favor the formation of particles with narrow size distribution. Thus, we chose ethanol/water mixed solvents as the solvent system.

Commercialized SiPs and CNFs were dispersed in the mixed solvent, and SiPs were deposited onto the CNF surface. During the solvent evaporation process, it was expected that the SiPs could prevent the agglomeration of CNFs. The interaction between the silica surface group and cellulose chain prevented immediate aggregation. To the best of our knowledge, there are no studies on incorporating cellulose fibers into hydrophobic polymers without surfactants or chemical modifications [3].

In this study, polypropylene (PP) was used as a polymer matrix. It is a widely utilized polymer with various advantages including low cost, superior transparency, good moisture barrier properties, and high recyclability compared to other polymers [16]. The composites, PP with fillers (SiPs, CNFs or SiP/CNF) were prepared by melt-blending in a twin-screw extruder. The effects of fillers and hybrid fillers in PP matrixes were investigated by the analysis of the matrixes’ morphological, chemical, thermal, and mechanical properties. The performance of pulverized SiPs and pulverized CNFs as filler in PP composites was also identified.

## 2. Materials and Methods

### 2.1. Materials

Polypropylene (PP), as a polymer matrix, was obtained from Japan Polypropylene Corporation, Tokyo, Japan (Polypropylene FY-6, MFR 2.5 g/10 min, CrI 59.80%). It has melting point of 162 °C, crystallization temperature of 104 °C and degradation temperature of 461 °C. Cellulose nanofibers (5 wt% aqueous dispersion, DP 650) were provided from Sugino Machine Limited, Toyama, Japan. The CNFs were produced by a super high-pressure water jet system. SiP powders, Sylosphere 200 (SS, diameter 3.0 µm) and Sylophobic 200 (SP, diameter 3.9 µm) were supplied from Fuji Silysia Chemical, Aichi, Japan. The ethanol (>99.5%) was purchased from Wako Pure Chemical Industries Ltd., Osaka, Japan, and reverse osmosis (RO) water was used throughout the experiments.

### 2.2. Filler Preparation Procedure

The compositions of the reinforced PP created by using as-prepared filler and hybrid filler samples prepared in this study are listed in Table 1. The required amount of SiPs and CNFs was added into the ethanol/water mixed solvents and stirred for one hour. The mixture was then evaporated by using a rotary evaporator (Eyela N-1110, Tokyo Rikakiki Co., Ltd., Tokyo, Japan). Subsequently, it was dried under vacuum overnight before use.

### 2.3. Composite Preparation

The desired amount of filler, hybrid filler, and PP polymer were fed into a twin-screw extruder with an ellipse-blade type screw L/D 0.5 (IMC-1979, Imoto Machinery Co., Toyama, Japan). The rotation speed, temperature, and residence time for melt-mixing were controlled at 40 rpm, 180 °C, and 5 min, respectively. The films were prepared using a hydraulic hot-press (IMC-180C, Imoto Machinery Co., Toyama, Japan) at 180 °C for 3 min under a pressure of 30 MPa, and then cooled at room temperature.

### 2.4. Characterization

The surface morphologies of SiPs were observed under a 3D laser scanning confocal microscope (LSCM) model VK-X 100 (Keyence Corporation, Osaka, Japan) under prescribed conditions of laser: red semiconductor laser, λ = 658 nm, 0.95 mW, and pulse width of 1 ns, using a depth composition procedure.

The morphology of the fractured composites and dispersion state of the hybrid filler in the PP matrixes were investigated using a scanning electron microscope equipped with energy dispersive spectroscopy (SEM-EDS) (JCM 6000, JEOL Ltd., Tokyo, Japan). SEM images were obtained at 15 kV accelerating voltage and the PP composite samples were fractured under liquid nitrogen. Each sample was deposited on carbon tape and carbon-coated for 90 s before observation.

The optical properties of the PP composite films were determined by UV–Vis spectra. A rectangular piece of each film sample (4 cm × 4 cm) was directly mounted between two spectrophotometer magnetic cell holders. The transmittance spectra of the films were measured at selected wavelength ranges from 190 to 1000 nm using a UV–Vis spectrophotometer (GENESYS 50, Thermo Fisher Scientific, Waltham, MA, USA). The optical properties of the PP and PP composite films were characterized by the transmittance of visible (660 nm) regions.

Chemical analysis was carried out using a Fourier Transform Infrared (FT-IR) spectroscope Nicolet iD7 ATR (Thermo Fisher Scientific, Waltham, MA, USA). Each sample recording consisted of 16 scans recorded from 4000 to 400 cm^−1^.

The PP composite films were cut into a rectangular shape of 40 mm × 5 mm × 0.5 mm. The mechanical properties of each PP composite were determined by an IMC-18E0 model machine (Imoto Machinery Co., Ltd., Kyoto, Japan) at a rate of 10 mm/min crosshead speed at 23 °C. Each measurement was carried out with five replicates.

The thermal properties of PP composites were characterized using an EXSTAR TG/DTA 7200 (SII Nanotechnology Inc., Chiba, Japan). The samples were scanned at a range of 30 to 550 °C at a constant heating rate of 10 °C/min under a continuous nitrogen flow rate of 100 mL/min.

## 3. Results and Discussion

### 3.1. Silica/CNF Filler Mechanism in PP Polymer

**Figure 1:**
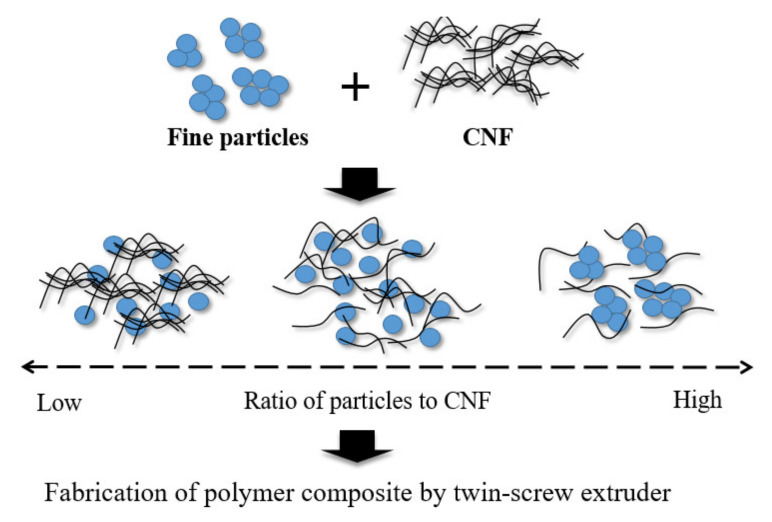
Interaction of fine particles and CNFs in ethanol/water mixed solvent.

Fine particles aggregated due to van der Waals forces or chemical bonds [17], and CNFs agglomerated due to strong fiber–fiber hydrogen bonding coupled with their polar nature, especially in a non-polar polymeric environment [18]. We hypothesized that the silica particles would adhere to the CNFs’ surface, which weakens the hydrogen bonds and prevents agglomeration. Figure 1 shows the interaction of the fine particles and CNFs if mixing in solvents. When the ratio of the fine particles is low, CNFs will agglomerate, and if too high, the fine particles will agglomerate. To further elucidate the respective mechanisms, SiPs were utilized and mixed with CNFs in ethanol/water mixed solvent. In this research, we only focus on the specific ratio of CNFs and SiPs first in order to investigate the basic interactions of the hybrid filler.

**Figure 2:**
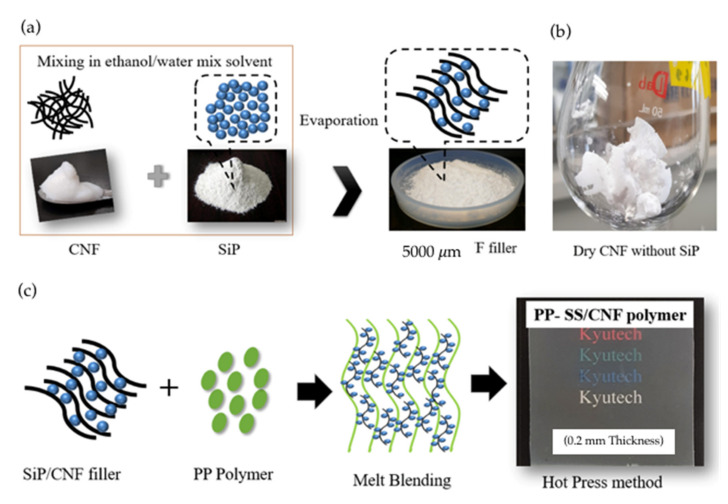
Images of SiP/CNF filler; (**a**) hybrid filler preparation, (**b**) agglomerated CNFs without SiPs and (**c**) hybrid filler mechanism in PP polymer.

Figure 2a,c shows the hybrid filler preparation in the ethanol/water mixed solvent and the possible hybrid filler mechanism in the hydrophobic polymer. Hydrophilic CNFs caused irreversible agglomeration during drying (Figure 2b), and due to the formation of additional hydrogen bonds between fibers, hydrophilic CNFs induced aggregation in the non-polar matrix [19]. Therefore, SiP powder was used to prevent the CNFs’ aggregation from occurring when incorporated into the hydrophobic polymer. From the observation of the hot-press film, the hybrid filler prevented the aggregation of CNFs in PP composites, and a good dispersion rate and transparency were achieved (Figure 2c).

Based on Figure 2a, the SiPs were mostly localized at each of the CNF fibers. Interaction between SiPs and CNFs was posited to be due to the presence of hydrogen bonds, due to the large surface areas and high amount of hydroxyl groups present in SiPs [20,21]. To further study the effect of hybrid fillers in hydrophobic polymer, PP polymer as a polymer matrix was melt-blended with fillers using a twin-screw extruder. Then, a hot-press sheet of the composite was prepared for further analysis. The chemical structures, thermal stabilities, and degree of substitution of MCC with different reaction temperatures and times were analyzed and investigated.

### 3.2. Surface Morphologies of the Hybrid Filler and PP Composite

**Figure 3:**
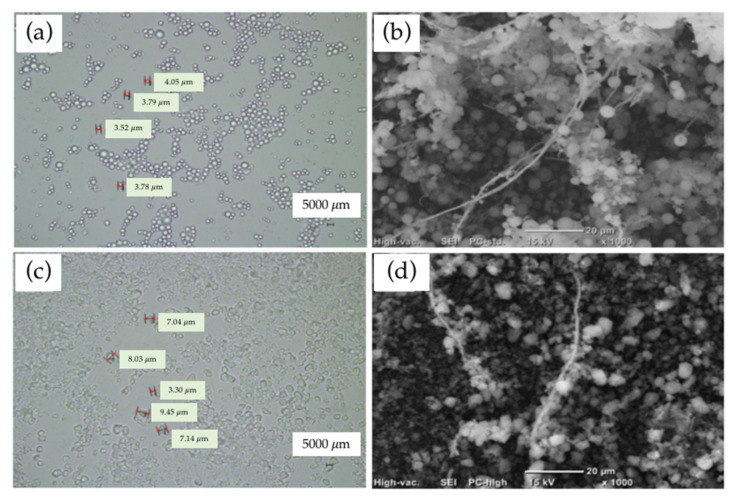
Morphological image of SiP particles: (**a**) SiP SS 50X, (**b**) SS/CNF 1000X, (**c**) SiP SP 50X, and SEM image of hybrid filler, (**d**) SP/CNF 1000X.

Figure 3a,c presents the surface morphologies of dried SiP SS and SP under a laser microscope with 50× magnification. A SiP SS is a single-distributed spherical silica particle and has a smooth surface with average size of 3–4 µm, having features such as high mobility and outstanding dispersity. Meanwhile, A SiP SP has a rough surface and irregular shape with an average size of 2–7 µm. According to the manufacturer, SiP SP demonstrates hydrophobic properties by chemically replacing the hydroxy groups on the silica surface with organic silicone compounds.

The structures of both hybrid fillers can be directly observed by using SEM. The SEM images in Figure 3b,d (SS/CNF and SP/CNF) illustrate that the SiPs were relatively homogeneously dispersed in the CNF matrix. It was verified that both types of SiPs were deposited predominantly on the surface of CNFs and prevented CNF agglomeration during the drying process. Both SiP SS and SP helped the dispersion of CNFs via a synergism effect. Silica not only offered a great surface area for coherence, but also provided aid in the dispersal of CNFs, which in turn reduced and minimized agglomeration. According to Sharip et al. [18], conventionally water-dispersed CNFs were employed to prepare a bio-nanocomposite, which required higher cost and energy. Hence, an ethanol/water mixed solvent method was used to address the setbacks of the conventional approach.

### 3.3. Optical Properties of the PP Composite with Fillers

**Figure 4:**
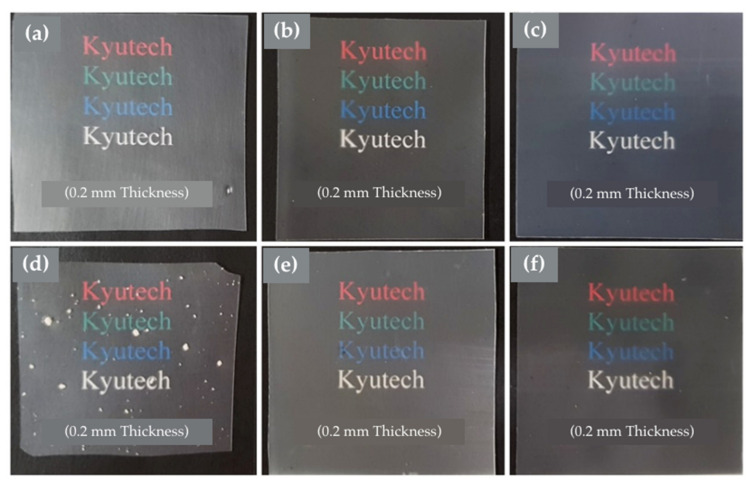
Digital images showing the transparency of (**a**) PP, (**b**) PP-8.75 SS, (**c**) PP-8.75 SP, (**d**) PP-1.25 CNF, (**e**) PP-SS/CNF, and (**f**) PP-SP/CNF.

The effect of different types of silica and CNFs on optical properties was elucidated. The PP composite was melt-blended through a twin-screw extruder. Figure 4 shows the PP composite after melt-blending and hot-pressing for analyses and the study of mechanical properties. Pristine PP (Figure 4a) acted as a reference. Figure 4b,c demonstrates the PP composite with SiP SS and SiP SP, while Figure 4e,f shows the PP-SS/CNF and PP-SP/CNF composites. Figure 4d presents the PP composite with only CNFs as the filler.

Based on observations, excellent dispersibility was noticed for the PP composite with SiPs and also the PP composite with SiPs/CNF. However, significant agglomeration or poor dispersion of CNFs can be observed in PP/CNF composites. This scenario was posited to be due to the different surface properties and the hydrophilic nature of CNFs. Inherently strong intra- and inter-molecular hydrogen bonds of CNFs led to a strong affinity to itself. Achieving good dispersion of fillers within polymer matrixes is the most critical factor in determining their resultant mechanical performance [22].

**Figure 5:**
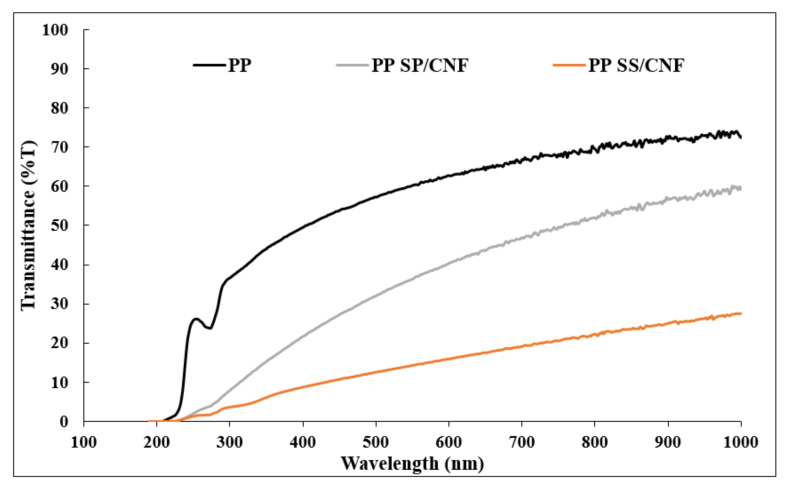
UV–vis transmittance spectra for PP and PP composite.

UV-vis spectroscopy analysis was carried out to further support the optical transparency results of the PP nanocomposites. Figure 5 shows the UV-vis transmittance spectra of the PP composite with the hybrid fillers, and the percentages of visible light transmittance values of the PP composite are summarized in Table 2.

The UV-visible spectra of PP nanocomposites were analyzed in the wavelengths between 200 and 800 nm. Based on data given in Table 2, the transmittance of plain PP was about 65.3 %T at 660 nm. After adding the hybrid fillers, the transmittance value was reduced. The transmittance efficiency decreased due to blocking by the hybrid filler. The dispersity of the reinforcement will affect the transparency of the nanocomposite. The transmittance level of the PP SP/CNF (44.6 %T) was higher than the PP SS/CNF (19.9 %T), even provided the same hybrid filler loading level. This scenario was due to the higher hydrophobicity of SP making it easier to incorporate with the hydrophobic PP polymer matrix.

### 3.4. Chemical Structure of Composite Films

**Figure 6:**
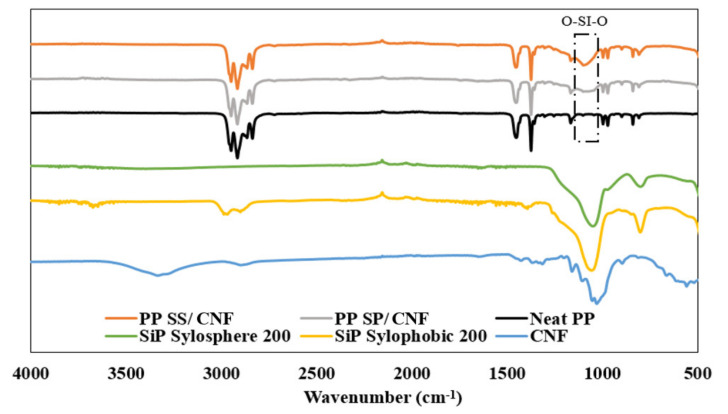
FTIR spectra of SiP Sylosphere 200, Sylophobic 200, CNF, and PP composites.

Functional group analysis of the PP and PP composites can be performed using FTIR spectroscopy. Figure 6 depicts the typical FT-IR spectra of pristine PP, SiPs, CNFs, and PP composite. The appearance of characteristic absorption bands of SiPs at 1054 and 1048 cm^−1^ were assigned to the siloxane Si–O–Si bonds for SiP SS and SP, respectively. Furthermore, transmittance peaks at 794 and 797 cm^−1^ indicated the presence of hydroxyl groups on the surface [23]. The C-H stretch vibration at 2966, 2898 and 1392 cm^−1^ were the typical characteristics of polyalkyl siloxane on the SiP SP as hydrophobic surface treatments [24].

For the CNFs, the broad peak at 3400–3300 cm^−1^ was attributed to the stretching vibration of O–H bonding from absorbed water molecules of the cellulose chains. This peak also included inter- and intra-molecular hydrogen bond vibrations in hydroxyl groups in cellulose I [25,26]. The peak at 2895 cm^−1^ was assigned to the C–H stretching vibration of all hydrocarbon constituent in polysaccharides and the peak at 894 cm^−1^ attributed to the β-glycosidic linkages of the cellulose chain [27].

Furthermore, the peaks at 2950–2835 cm^−1^ were contributed by C–H stretching vibrations while peaks at 1450 and 1376 cm^−1^ were assigned to CH_2_ and CH_3_ bending vibration in neat PP [28]. Further, the transmittance peak located at 840 cm^−1^ was assigned to C–CH_3_ stretching vibration [29]. This peak is a typical characteristic of PP polymers.

New transmittance peaks of the PP nanocomposites at 1087 and 1076 cm^−1^ of the asymmetric vibration of O–Si–O bonds indicated that PP with SS/CNF and SP/CNF contained silica fillers. In addition, increasing peak intensity at 806 cm^−1^ indicated stretching vibrations of Si–O. From the FT-IR spectra, it is confirmed that the hybrid fillers did not damage the composite materials via denaturing through the extrusion process. However, the characteristic bonds of the C–O group in CNFs were undetected due to the overlap with the O–Si–O band [30].

### 3.5. Mechanical Properties of PP Composite

**Figure 7:**
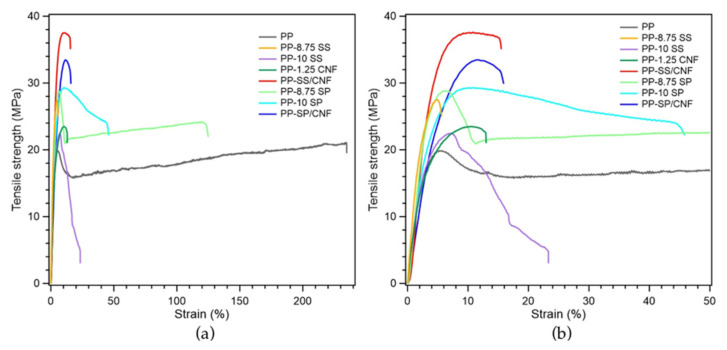
Mechanical properties of (**a**) stress-strain curves of PP composites and (**b**) an enlarged graph.

Mechanical properties such as tensile strength and elongation are often used to measure the strength and elasticity of composite films. The representative tensile stress-strain curves of pure PP and its composites are shown in Figure 7 and their key mechanical properties are summarized in Table 3. The fillers incorporated into composite films significantly affected the mechanical properties of the composites due to the specific surface area and dispersibility of each filler. Improved mechanical properties can be achieved through an improved interface between the filler and polymer matrix [31].

**Figure 8:**
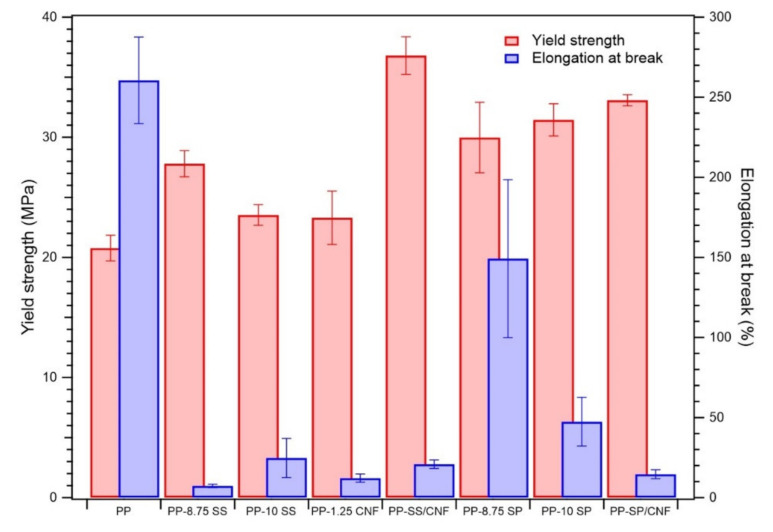
Mechanical properties of PP composites: bar charts of elongation at break and yield strength.

Figure 7 and Figure 8 and Table 3 also show the effect of filler on the tensile strength at yield of PP composites compared to pristine PP polymer. The yield strength of PP increased after melt-blending with filler or hybrid filler. This scenario was due to the addition of filler into the matrix, which reduced the available free spaces and hence increased the stiffness of the composite. The filler linked the matrix together, leading to enhanced interaction between the reinforcement and the matrix. Once the load has been applied, the stress can easily be transferred from the polymer matrix to the reinforcing materials, thus improving the tensile strength of the polymer composite [32].

The tensile strength at yields of PP-8.75 SS and PP-10 SS was 27.80 and 23.53 MPa, respectively. Increasing the by-weight percentage of SS led to inferior mechanical properties. The agglomeration extent increased with the increase of nanofiller content and reduction of nanofiller size. When compared to SP, the particle size of SS is smaller overall, so aggregation has a higher chance to occur in PP/SS composites. Agglomeration decreased the effectiveness of nanoparticles in polymer matrix, which lastly resulted in the poor properties of the samples.

However, increasing the by-weight percentage of SP from 8.75 to 10 increased the tensile strength. The irregular shape and slightly larger particle size of the filler produced different properties. The surface roughness and irregular shape enhanced the interfacial properties between the matrix and reinforcement, such that the tensile strength still increased after the addition of 10 wt.% of SP. Both PP-SS/CNF and PP-SP/CNF samples depicted excellent yield strengths, which were 36.81 and 34.06 MPa, respectively. The synergy of the hybrid filler occurred due to the possibility of physical interaction between the functional groups of SiPs and CNFs. This functional group built the interfacial surface compatibility and increased the dispersibility between the CNFs and PP matrix. In addition, the homogeneity and matrix particle interactions were improved after adding the hybrid filler. Compared to the pristine PP (21.2 MPa), the tensile strength at yield of PP-SS/CNF and PP-SP/CNF samples showed 73.5% and 60.3% increments, respectively.

Furthermore, the elongation at break of PP composites was sharply reduced by the addition of filler due to the stiffening effect of SiPs and CNFs on the PP composite [31]. The presence of a rigid interface between SiPs and CNFs and the PP matrix decreased the deformability of the PP matrix, which led to more rigid and stiffer composites [28,33]. The PP-8.75 SP (201.70%) composites exhibited the highest elongation at break. SP is a hydrophobic filler, which can enhance the interfacial interaction between the filler and PP matrix and generate a stronger interfacial bonding. However, further study needs to be carried out to investigate the unexpected elongation behavior of sample PP-8.75 SP compared to other polymer composites.

### 3.6. Morphology of the Fracture Surface of PP Composites

**Figure 9:**
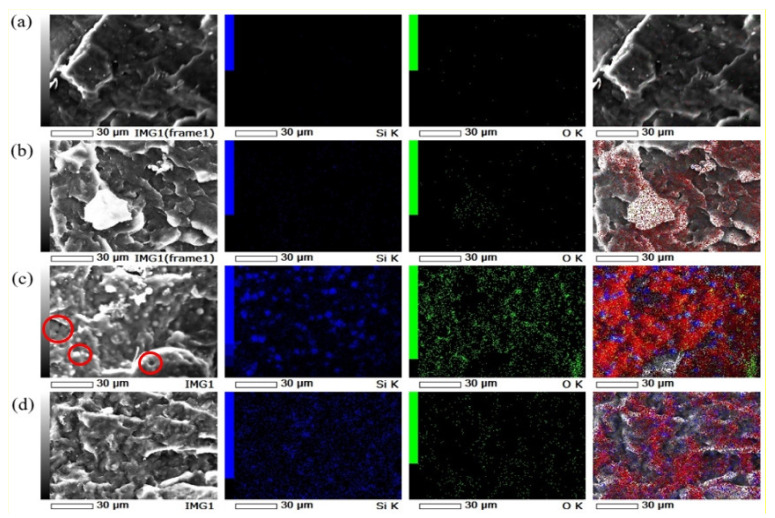
SEM images mapped with EDS analysis for distribution of oxygen (O) element represents CNF and silica (Si) element represent SiP: (**a**) PP polymer, (**b**) PP-1.25 CNF, (**c**) PP SS/CNF and (**d**) PP SP/CNF.

PP composites were further analyzed with SEM-EDS to observe their cross-sectional fracture surface. First, each sample was submerged in liquid nitrogen for 5 min to ensure it was completely frozen. It was then removed from the liquid nitrogen and immediately snapped in half. The sample was dried to remove excess water. After freeze-fracture, the cross-section of the sample was carbon-coated and observed. Figure 9 depicts the SEM-EDS micrograph and quantitative analysis of elements present at the white spot. The elements are oxygen (O) and silica (Si). The SEM image also shows the typical fracture surfaces of neat PP and PP composite samples. This characterization technique can be used to identify the hybrid filler distribution in the polymer composite as well.

In Figure 9a, the rough surface observed on neat PP indicated the typical characteristic of the elastic behavior of PP [34] and the element mapping of Si and O was almost completely black in colour. In addition, it can be observed that CNFs were agglomerated in the polymer composite (white circle) in the EDS analysis and also SEM morphology, which is supported by the study of Yasim-Anuar et. al. [35]. According to the respective literature, the distribution of oxygen (O) represents CNFs (Figure 9b).

Moreover, the EDS images showed the samples PP SS/CNF and PP SP/CNF in Figure 9c,d to consist of uniform white spots of O and Si, which further confirm the good dispersion of the hybrid filler in the polymer composite. The oxygen element of PP nanocomposites increase compared with PP/CNF is due to Si-O-Si bonding formed after blending. These results are in line with the FT-IR assignment in the previous section.

Further, the SEM images present a uniform distribution of the hybrid fillers in a PP composite surface. Sample PP SS/CNF showed that the particulate fillers with a spherical and fibrous shape (red circle) were found in the composite, suggesting the applied stress effectively transferred to the filler from the polymer matrix by scattering the energy during crack propagation [36]. While on the sample PP SP/CNF, interconnected nanofibers formed with rough surfaces and irregular shapes were observed. SiP SP exhibited better interfacial bonding strength and filler dispersion in the PP composite. These results indicate robust interfacial adhesion between the PP matrix and filler due to their hydrophobicity compatibility.

### 3.7. Thermogravimetric Analysis (TGA) of PP Composite

**Figure 10:**
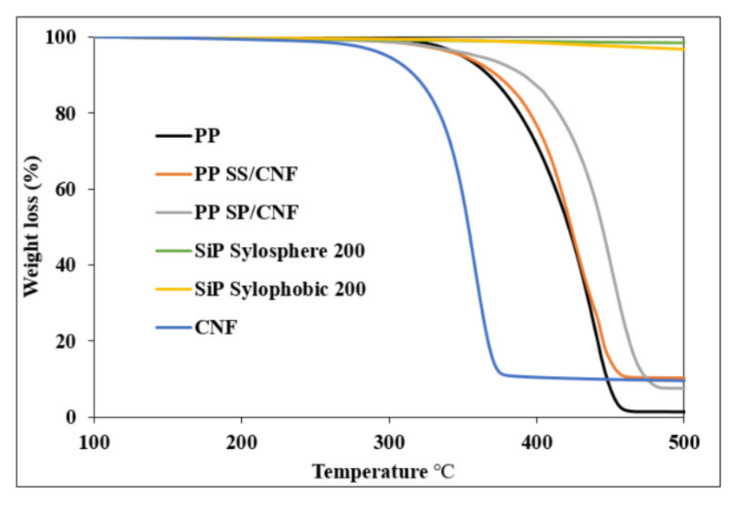
TGA curves of PP, SiP, CNF, PP SS/CNF, and PP SP/CNF.

Figure 10 shows the thermal stability of the neat PP, SiPs, CNFs, and PP composite. The TGA results show the neat PP and PP composite degradation in a single step of degradation. SiP SP had weight loss at 420 °C, attributed to the thermal decomposition of the functional group of polyalkyl siloxane. Above 450 °C, there was no weight loss observed for either SiP sample, indicating residual SiPs. Further, SiPs have high thermal stability at temperatures up to 800 °C [37]. For the CNF sample, weight loss started from 270 °C due to the thermal decomposition of cellulose [38]. The TGA result also showed the neat PP almost degraded without any char formation, with the residual of the original sample mass being only 1.3%.

Compared to the neat PP, the decomposition temperature of the PP composite increased significantly with the addition of hybrid filler, indicating that SiPs hindered thermal decomposition of PP composite. The degradation depends on the particle encapsulation and extent of interaction with matrix. Improvement in the thermal stability of PP composites was posited to be due to the excellent dispersion of the hybrid filler in the polymer matrix. Due to the stable thermal properties of the hybrid filler, chain scission process occurred at a higher temperature compared to pristine PP polymer [39]. Additionally, the weight residue at 500 °C of the PP composite showed the highest for sample PP SS/CNF at 10.28% residue, followed by CNF at 9.54% residue, and PP SP/CNF at 7.53% residue. The char yield increase after melt-blending with filler is attributed to the increased interactions between the filler and the matrix which resulted in the onset of thermal degradation.

**Figure 11:**
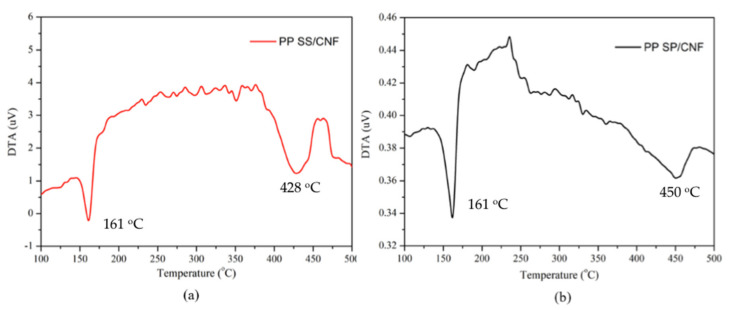
DTA curves of (**a**) PP SS/CNF, and (**b**) PP SP/CNF.

Additionally, the DTA curves of the discussed composite PP SS/CNF and PP SP/CNF show that during their decomposition, endothermic reactions occurred (Figure 11). The DTA curve exhibited two endothermic events. The first is attributed to Tm at around 161 °C, corresponding to the melting of the crystalline phase in the studied polymer (PP). Other endothermic peaks were observed with decomposition maxima at 428 and 450 °C in higher temperature ranges. The thermal decomposition of PP SP/CNF is higher than PP SS/CNF.

## 4. Conclusions

An environmentally benign ethanol/water mixed solvent method was used to prepare a PP-SiP/CNF composite. The method applied in this work is very convenient, requires less energy, and sidesteps chemical modification. It is noted that a synergistic effect of the hybrid filler occurred in this composition, showing a significant enhancement of the dispersion of CNFs in the hydrophobic polymer. Morphology results confirmed the deposition of SiPs onto the CNF surface, resulting in good dispersion of the hybrid filler in the PP matrix and preventing the agglomeration of CNFs. The incorporation of filler into the PP matrixes significantly increased the mechanical properties of the composites. Sample PP-SCNF exhibited higher tensile strength at 36.8 MPa (increments 73.55%) compared to the neat PP. However, its elongation sharply reduced due to the stiffening effect of the filler. The thermal stability of PP composites was improved by the incorporation of fillers as a physical barrier. The hybrid filler exhibited synergistic effects, especially for tensile strength, and proved to be more effective than single-filler systems. The simplicity of this strategy can be applied to other fillers such as metal oxide and graphene oxide to prepare hybrid fillers for designing polymer composites, especially to enhance the mechanical properties and thermal stability of a product.

## Figures and Tables

**Table 1:** Sample composition of PP and filler.

Sample	PP (wt%)	Sylosphere (wt%)	Sylophobic (wt%)	CNF (wt%)
PP	100	-	-	-
PP-8.75 SS	91.25	8.75	-	-
PP-10 SS	90	10	-	-
PP-1.25 CNF	98.75	-	-	1.25
PP-SS/CNF	90	8.75	-	1.25
PP-8.75 SP	91.25	-	8.75	-
PP-8.75 pul.SP	91.25	-	8.75	-
PP-10 SP	90	-	10	-
PP-SP/CNF	90	-	8.75	1.25

**Table 2:** Optical properties of PP and PP composite.

Sample	Thickness (mm)	%T (Transmittance) (660 nm)
PP	0.255	65.3
PP SS/CNF	0.446	17.9
PP SP/CNF	0.356	44.6

**Table 3:** Mechanical properties PP composites.

Sample Code	Tensile Strength at Yield (MPa)	Tensile Elongation at Break (%)
PP	20.78 ± 1.06	260.50 ± 27.03
PP-8.75 SS	27.80 ± 1.09	7.30 ± 1.10
PP-10 SS	23.53 ± 0.86	24.71 ± 12.20
PP-1.25 CNF	23.30 ± 2.22	12.17 ± 2.54
PP-SS/CNF	36.81 ± 1.56	20.87 ± 2.68
PP-8.75 SP	29.98 ± 2.94	149.20 ± 49.36
PP-10 SP	31.45 ± 1.34	47.40 ± 15.21
PP-SP/CNF	33.08 ± 0.47	14.57 ± 2.77

## Data Availability

The data presented are contained within the article.
